# Study of shear resistance and anisotropy of layered shales

**DOI:** 10.1371/journal.pone.0313134

**Published:** 2024-12-05

**Authors:** Min Gao, Bin Gong, Zhengzhao Liang, Shanpo Jia, Xianhui Feng

**Affiliations:** 1 Engineering Research Center of Underground Mine Construction, Anhui University of Science and Technology, Huainan, P.R. China; 2 Institute of Unconventional Oil and Gas, Northeast Petroleum University, Daqing, P.R. China; 3 College of Engineering, Design and Physical Sciences, Brunel University London, London, United Kingdom; 4 State Key Laboratory of Coastal & Offshore Engineering, Dalian University of Technology, Dalian, P.R. China; 5 School of Civil and Resources Engineering, University of Science and Technology Beijing, Beijing, China; University of Science and Technology Beijing, CHINA

## Abstract

Characterizing anisotropy remains challenging in rock mechanics. Particularly, the strengths and failure patterns of layered shales under shear load are significantly anisotropic mainly because of the bedding planes. Meanwhile, understanding the creation and propagation of shear fractures is critical for drilling, mining, tunnelling, exploitation of shale gas, *etc*. In this study, the shear resistance of layered shales is comprehensively investigated based on the direct shear tests numerically. The results show that the shear parameters are greatly affected by the anisotropy induced by the normal stress and orientation of bedding planes; the shear strength, cohesion and internal friction angle generally increase with the growth of bedding plane orientation. Furthermore, three shear failure patterns are summarized, *i*.*e*., (1) the shear failure along bedding planes; (2) the shear failure crossing bedding planes; (3) the combination of tensile failure along bedding planes and shear failure crossing bedding planes. Besides, the empirical fitting formula characterizing the shear strength of layered rocks under triaxial compression is provided, and the modified Mohr-Coulomb criterion reflecting rock anisotropy is proposed.

## 1 Introduction

Anisotropy is recognized as one main mechanical characteristic of layered rocks [1–3]. For layered rocks, the deformation and strength obviously show anisotropic characteristics because of the existence of bedding planes. Meanwhile, shear failure is a critical failure and instability mode in rock mechanics. Shear stress concentration often occurs at the interface between bedding planes and rock media, which may lead to shear slip failure of layered rock masses that are commonly observed in rock engineering. Furthermore, the anisotropic characteristics of shear failures of layered rock masses have a significant impact on the safety and stability evaluation of layered rock engineering [4–6].

The related studies have been conducted to understand the material anisotropy influenced by bedding planes. In principle, the mechanical parameters, such as compressive/tensile strength, deformation, failure mode and interface friction, are the focuses of the most investigations since these are the pivotal indices for the stability analysis for engineering projects during design, construction and management [7–11]. Clearly, many experimental and numerical approaches have been adopted to investigate the anisotropic material parameters of layered rocks. The uniaxial & triaxial compressive experiments are common approaches to obtain the anisotropic parameters of layered rocks. The reported data indicates that the compressive strength of layered rocks decreases first and then increases with the bedding orientation increasing, which displays a typical ‘U’ shaped curve. However, with the growth of lateral pressure in triaxial compressive test, the anisotropy of compressive strength is basically weakened. Moreover, the anisotropy of deformability and fracturing mechanism were measured in the related uniaxial and triaxial compression tests [12–17]. The anisotropy of tensile strength and corresponding failure mechanisms of layered rocks was investigated by the Brazilian tests using rock discs [18–24]. The current data suggests that the anisotropy of the tensile strength and corresponding failure mechanisms highly depends on the loading angle between loading axis and bedding orientation. Although the anisotropic material parameters of layered rocks have drawn much attention and a series of valuable results have been obtained, the anisotropic features of shear parameters and shear failure mechanisms of layered rocks are relatively less reported.

The methods of investigating shear mechanical properties and failure mechanisms of layered rocks can be generally divided into two types, *i*.*e*., direct shear test and indirect compression test. Actually, the shear mechanical properties and constitutive models of jointed rock masses have been studied by extensive direct shear tests, and the influence of different scales of joint roughness on the shear behavior has been quantified [25]. For instance, Moradian *et al*. (2010) [26] analyzed the effect of joints on the shear mechanical parameters of layered rock masses through shear test in the laboratory. Simultaneously, the direct shear tests were also carried out to explore the anisotropy of shear strength or other mechanical parameters and the corresponding mechanisms of layered rocks [27, 28]. The data shows that the shear failure can be classified into three types, *i*.*e*., sliding failure crossing bedding planes, sliding failure along bedding planes, and sliding failure crossing bedding planes combined with tensile splitting along bedding planes. Attewell and Sandford (1974) [29] obtained the variation of cohesion and internal friction angle associated with the bedding plane orientations using the triaxial compression test. Similar to the uniaxial compressive strength, the changes of cohesion and internal friction angle of layered rock masses also presented the ‘U’ shaped trend. Based on this trend, they proposed the empirical formula of cohesion and internal friction angle of the layered rock masses. Zhang *et al*. (2010) [30] proposed the empirical formulas for the changes of cohesion and internal friction angle affected by the bedding plane orientation of layered rock masses on the basis of the principle of single weak plane. This empirical model was numerically implemented by combining the Mohr-Coulomb criterion and the finite difference method. Nova (1980) [31] theoretically analyzed the failure mechanical properties of transversely isotropic rock under triaxial compression and obtained the analytical solution of cohesion and internal friction angle variation with different bedding orientations.

In this study, the failure behavior of layered rock masses under direct shear test has been comprehensively investigated numerically. The anisotropic characteristics of shear strength parameters were discussed and analyzed by comparing with the related experimental results. Meanwhile, the shear strength parameters of layered rock masses under triaxial compression test were obtained by the theoretical analyses and numerical simulations. The shear failure mechanisms of layered rock masses were analyzed by comparing the results under direct shear test and triaxial compression test.

## 2 Simulation of direct shear test on layered rocks

### 2.1 Numerical model and material properties

**Fig 1:**
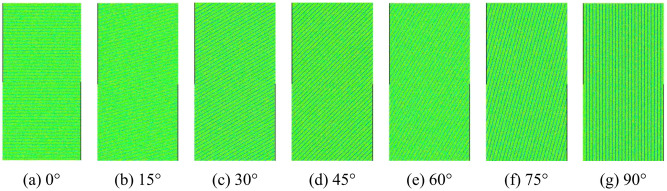
Numerical models of layered rocks for direct shear test.

**Fig 2:**
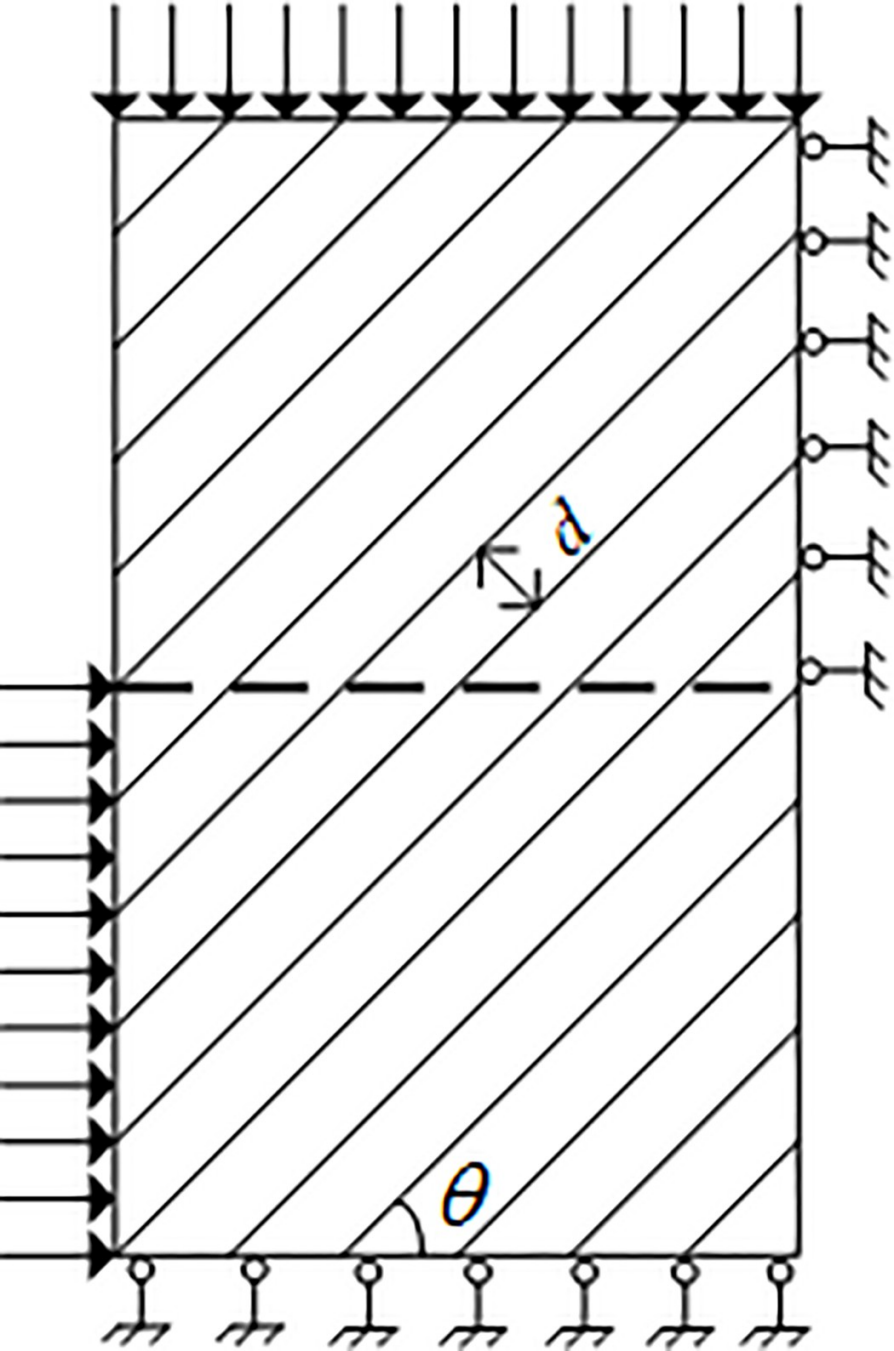
Loading diagram of the layered rock samples suffering direct shearing.

The numerical specimens of layered rocks with various bedding orientations are established by the two-dimensional rock failure process analysis (RFPA2D) method to conduct the direct shear tests, as shown in Fig 1. The principles of RFPA have been demonstrated by [32], and its effectiveness has been verified by the related studies [33–36]. The layered rock specimens are composed of rock matrix and joint material. Table 1 lists the mechanical parameter values which are determined by the ‘trial and error’ approach described by [24]. In rock engineering, the mechanical parameters, such as cohesion and internal friction angle, can be determined by the confined compression, tensile and shear tests. The diagram of layered rock samples suffering direct shearing is displayed in Fig 2. The bottom of the numerical specimens is constrained along the normal direction, and the top surface of the numerical specimens is subjected to the normal stress from 2.5 MPa, 5 MPa, 7.5 MPa to 10 MPa. Simultaneously, the upper right side of the specimens is constrained along the horizontal direction, while the lower left side of the samples is subject to the horizontal displacement load with the rate of 0.001 mm/step.

**Table 1:** Mechanical parameters of the layered rock samples.

Material	Compressive Strength (MPa)	Elastic Modulus (MPa)	Poisson’s Ratio	Inhomogeneous Coefficient
Rock material	933	90,815	0.30	4
Joint material	311	26,689	0.35	3

### 2.2 Principle of direct shear test

**Fig 3:**
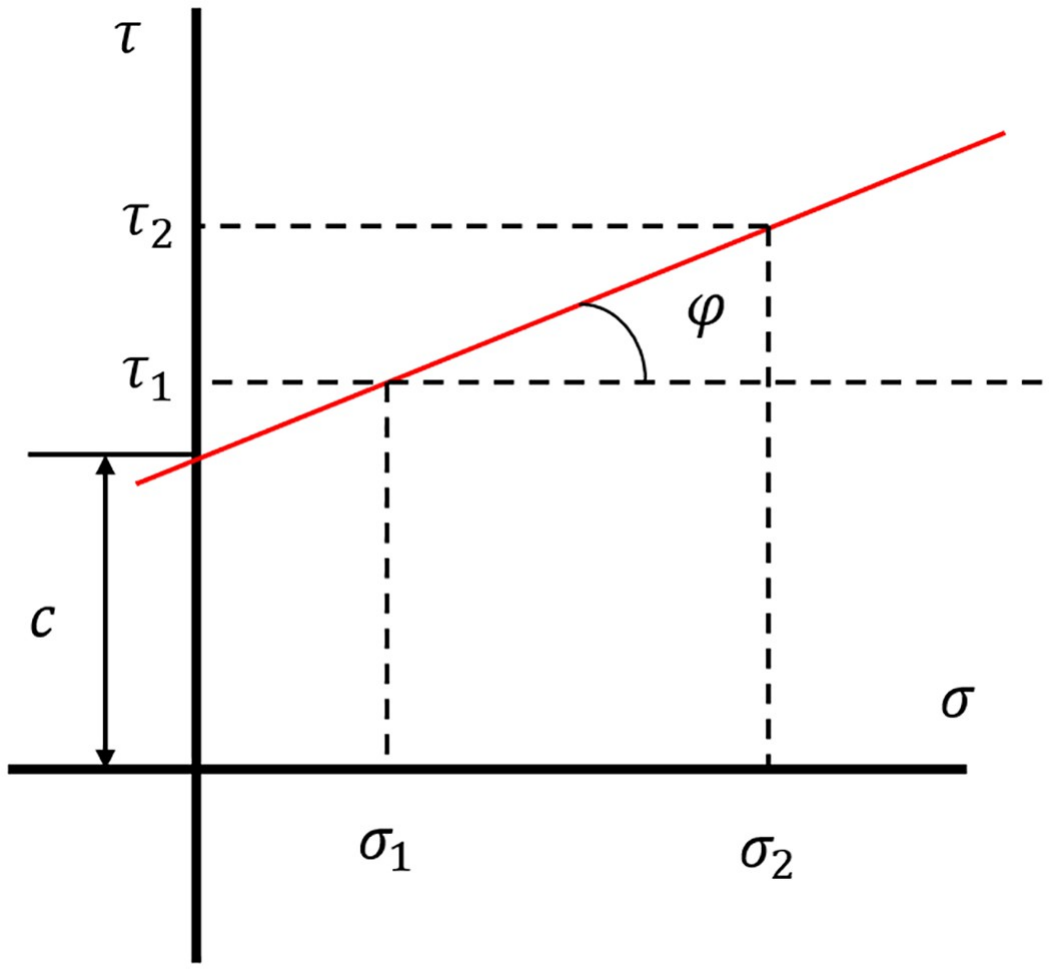
Mohr-Coulomb envelope.

On the basis of the Mohr-Coulomb criterion, the relationship between normal stress and shear stress of the specimens can be described by Eq (1):

$$\tau =\sigma_{n}tan\varphi +c$$ (1)

where *φ* and *c* represent the internal friction angle and the cohesion of the shear surface, respectively. Fig 3 shows the linear Mohr-Coulomb stress state in the shear region, where *σ*_1_ and *σ*_2_ are the normal stresses; *τ*_1_ and *τ*_2_ are the related shear stresses, respectively. Theoretically, if the two stress states are given, the cohesion and internal friction angle of the specimen can be gained.

## 3 Numerical results and comparative analyses

### 3.1 Failure modes and mechanisms

**Fig 4:**
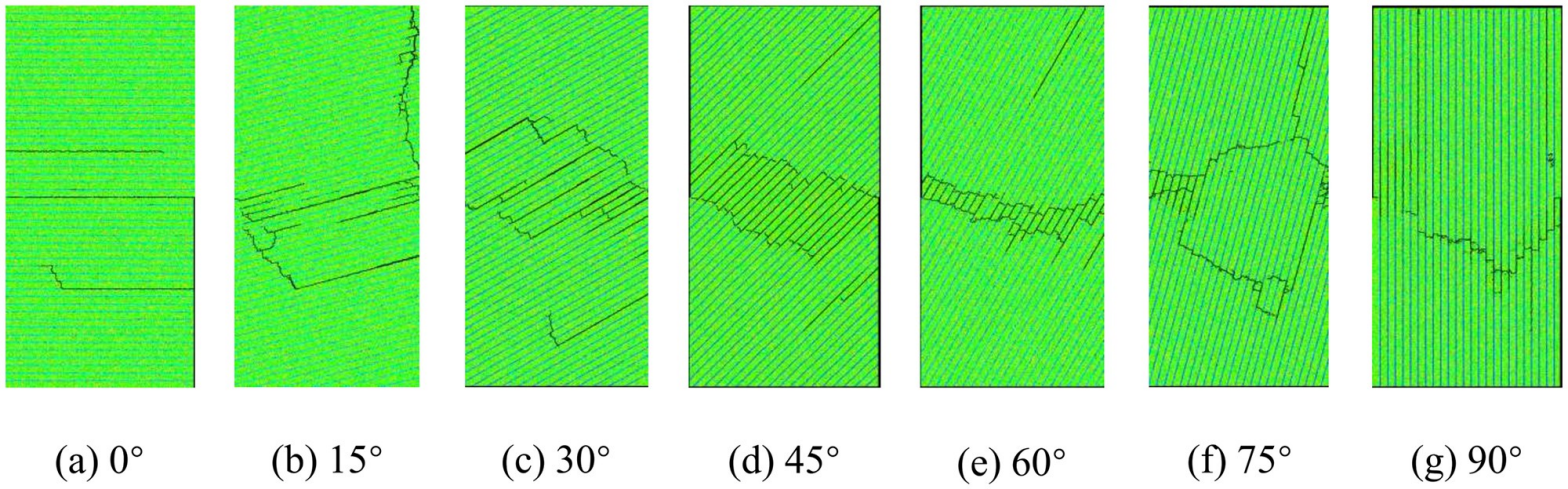
Failure patterns of layered rock samples suffering direct shearing when *σ*_*n*_ = 2.5 MPa.

Fig 4 displays the failure modes of layered rock samples with various bedding orientations in the numerical direct shear test when *σ*_*n*_ = 2.5 MPa, from which we can see that the failure patterns of the rock samples suffering direct shearing are obviously different, indicating the typical anisotropic characteristics. For the sample with the bedding orientation of 0°, three shear failure surfaces occur inside the specimen and are parallel to the bedding planes. For the specimens with the bedding orientation between 15° and 60°, the single or multiple failure surfaces do not appear inner the specimens. However, a fracture zone forms at the middle of the samples. As the bedding plane orientation increases, the fracture zone shrinks to the central area of the specimens. When the normal stress keeps constant during the numerical test, with the growth of the horizontal load, the specimens are subject to shear fracture along multiple bedding planes firstly. However, the shear cracks don’t propagate and coalesce inside the whole specimens. Then, the rock material between the cracked bedding planes suffers tensile cracks, and they gradually coalesce with the fractured bedding planes. The failure pattern of these specimens is the combined shear-tensile failure. Furthermore, the failure pattern of the sample with the bedding orientation of 75° is more complex. Two failure surfaces form in the specimen, and the failure planes are relatively rough. During the formation process of the two failure planes, many shear fractures occur along the bedding planes, while the cracks are relatively small. The rock material between the cracked bedding planes suffer shear cracking inside the specimen. For the specimen with the bedding orientation of 90°, the rock material suffers shear cracking at the middle part of the sample, and a single failure plane forms at the middle of the specimen.

Therefore, the failure patterns of the layered rock samples under direct shear can be classified into three types, *i*.*e*., shear failure along the bedding planes, composite failure with bedding planes shear sliding and rock material tensile fracture, and shear failure of rock material. Wang *et al*. (2017) [37] reported the experimental results of layered rock masses suffering direct shearing. By comparing the numerical results with their experimental data, it can be concluded that the failure modes of layered rock specimens with varying bedding plane inclinations under direct shear test are basically the same. The failure patterns of the experimental results can also be classified into three categories, which can verify the accuracy of the numerical simulations in this study.

The failure mechanisms of the layered rock samples under direct shearing can be divided into three categories on the basis of the failure patterns obtained by the numerical experiment. (1) When the orientation of bedding planes is 0°, the failure mode of the layered rock sample is shear failure along the bedding plane. In this scenario, the shear strength of the layered rock sample mainly depends on the mechanical resistance of bedding planes; (2) When the orientation of bedding planes is close to 90°, the failure pattern of the layered rock specimen is shear failure of rock material cross the bedding planes, and the failure plane is basically vertical to the bedding planes. In this scenario, the shear strength of the layered rock specimen mainly relies on the mechanical resistance of the rock material; (3) When the orientation of bedding planes is between 15° and 60°, the failure pattern of the layered rock samples is the compound failure including shear failure of bedding planes and tensile failure of rock material. In this scenario, the shear strength of the layered rock specimen mainly relies on the mechanical resistance of the rock material and bedding planes.

### 3.2 Correlation between shear stress and tangential deformation

**Fig 5:**
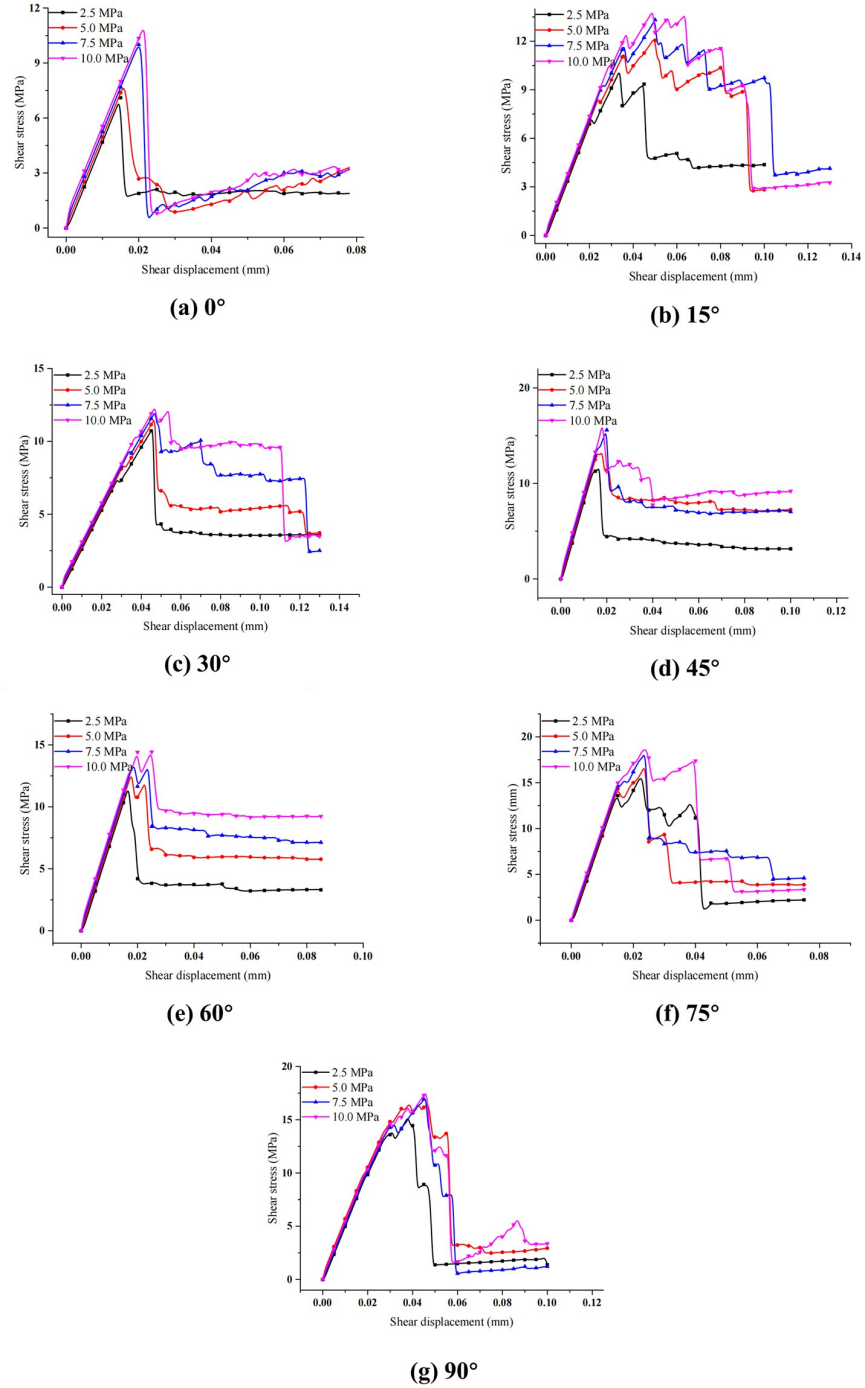
Shear stress vs. shear displacement of the layered rocks under various normal stresses.

Fig 5 shows the relationship between shear stress and tangential deformation of layered rock samples with various bedding plane orientations subject to varying normal stresses, from which we can see that the curves present obvious slip softening stage. For the layered rock samples with various bedding plane orientations, the shear stress increases almost linearly with shear deformation at the initial loading stage of the curves. For the layered rock specimens with the same bedding plane orientation, the curves of shear stress changing with shear displacement are basically consistent under different normal stress loads before the pre-peak shear strength. For the sample with the bedding plane orientation of 0°, the shear stress drops suddenly after peak strength, which may be related to the failure modes of layered rock samples. The failure mode of the specimen with the bedding plane orientation of 0° is shear fracture along the bedding planes, and the failure plane is along the preset shear failure plane. With the growth of horizontal displacement load, the specimen shows shear failure suddenly along the bedding surfaces, then the curve of shear stress changing with shear deformation drops abruptly after the peak strength. This phenomenon suggests that when shear slip occurs along the bedding planes, the cohesion of these planes is abruptly lost, which also accounts for their weak bond strength. Simultaneously, we can see from Fig 5 that when the normal stress is bigger, the instantaneous drop amplitude of post-peak shear stress would be larger.

For the specimens with the bedding plane orientation of 75° and 90°, the growth rate of shear stress gradually slows down with the shear deformation increasing under varying normal loads. The shear stress on these curves presents a wave increase, and the shear stress drops gently after the peak shear strength. These characteristics of the curves may be related to the failure patterns of the samples. The main failure of the samples is shear facture of rock material before the specimens reach the shear strength. The failure surface is relatively unsmooth and shows a certain serrated shape because of the high strength of the rock matrix, resulting in the growth of the sample shear stress in a fluctuation pattern before the peak shear strength.

For the specimens with the bedding plane orientation of 15°, such change of shear stress vs. shear displacement curve is more significant. The failure pattern of the sample with the bedding plane orientation of 15° is the combined failure of shear and tension. The vertical splitting cracks throughout the weak beddings appear at the upper right boundary of the sample. When the specimen reaches the peak shear strength, the shear stress drops more slowly in a fluctuant way. For the specimens with the bedding plane orientation between 30° and 60°, shear stress almost linearly increases with the growth of shear displacement before the specimens reach the shear strength. The growth rate of shear stress drops down with the shear displacement increasing when the stress of the specimens approaches the shear strength. After reaching the peak shear strength, with the shear displacement increasing, the shear stress drops rapidly to the residual shear strength. The slope of the shear stress-shear displacement curve decreases rapidly, and the sample presents the classical brittleness features. At this stage, shear strength of the rock specimens shows obvious slip-softening features, but the characteristics for the specimens with different orientations are slightly different.

## 4 Theoretical analysis of anisotropic shear strength

### 4.1 Theoretical analysis

Heng *et al*. (2014, 2015) [27, 28] established the theoretical model of direct shear destruction analysis of stratified rock mass and analyzed the direct shear strength of shale anisotropy. The theoretical analysis model was developed based on three basic assumptions: (1) the torque caused by the eccentric load in the direct shearing process can be ignored; (2) there is only shear force along the preset shear zone direction; (3) the shear stress at the sheared layer is proportional to the relative deformation of the upper and lower connected non-shear layers, *i*.*e*., the shear stress of the shear layer is assumed to be in the elastic state.

**Fig 6:**
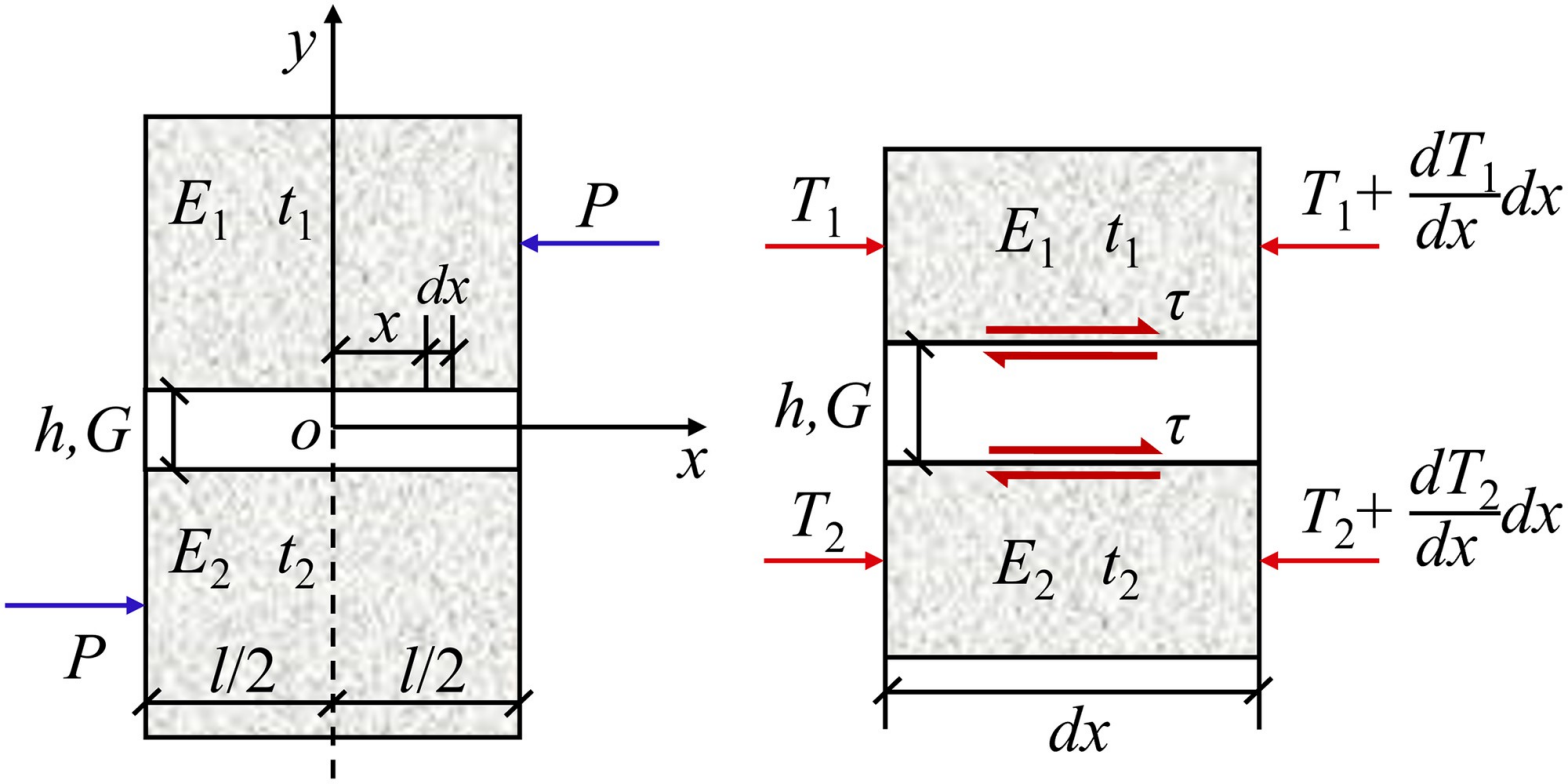
Shear layer and mechanical analyses along the *x* direction.

To simplify the analysis, it is assumed that the force lines exist only at the shear layer to solve the shear stress field of the shear layer, as shown in Fig 6. Taking the micro element to analyze, the equilibrium equation for the upper non-shear layer and the lower non-shear layer is as follows:

$$\left.\begin{array}{c} -\frac{dT_{1}}{dx}+\tau =0\\ -\frac{dT_{2}}{dx}-\tau =0 \end{array}\right\}$$ (2)

where *T*_1_ and *T*_2_ are the tangential forces of the upper & lower non-shear layers of micro elements, respectively. In Fig 6, *E*_1_, *E*_2_, *t*_1_ and *t*_2_ are the elastic moduli and thicknesses of the upper & lower non-shear layers, respectively. Besides, *P* is the shear force per unit width; *h* and *l* are the thickness and length of the shear layer, respectively; *G* is the shear modulus of the shear layer in the *xy* plane.

According to the basic hypothesis, the elastic constitutive relation and the geometric deformation relation of shear layer, *T*_1_, *T*_2_ and *τ* can be obtained as follows:

$$T_{1}=\frac{P}{2}\left(\frac{sinh\lambda x}{\text{sinh}\left(\frac{\lambda l}{2}\right)}+\frac{E_{2}t_{2}-E_{1}t_{1}cosh\lambda x}{E_{1}t_{1}+E_{2}t_{2}cosh\left(\frac{\lambda l}{2}\right)}+\frac{2E_{1}t_{1}}{E_{1}t_{1}+E_{2}t_{2}}\right)$$ (3)

$$T_{2}=\frac{P}{2}\left(-\frac{sinh\lambda x}{\text{sinh}\left(\frac{\lambda l}{2}\right)}-\frac{E_{2}t_{2}-E_{1}t_{1}cosh\lambda x}{E_{1}t_{1}+E_{2}t_{2}cosh\left(\frac{\lambda l}{2}\right)}+\frac{2E_{2}t_{2}}{E_{1}t_{1}+E_{2}t_{2}}\right)$$ (4)

$$\tau =\frac{\lambda P}{2}\left(\frac{cosh\lambda x}{\text{sinh}\left(\frac{\lambda l}{2}\right)}+\frac{E_{2}t_{2}-E_{1}t_{1}sinh\lambda x}{E_{1}t_{1}+E_{2}t_{2}cosh\left(\frac{\lambda l}{2}\right)}\right)$$ (5)

where $\lambda =\sqrt{k(\frac{1}{E_{1}t_{1}}+\frac{1}{E_{2}t_{2}})}$, *sinh*, *cosh* and *coth* are the related mathematical symbols of the hyperbolic functions and *k* is a scale factor.

In the direct shear numerical test in this study, *E*_1_ = *E*_2_ = *E*, *t*_1_ = *t*_2_ = *t*. Hence, the Eq 5 can be simplified as follows:

$$\tau =\frac{\lambda P}{2}\frac{cosh\lambda x}{\text{sinh}\left(\frac{\lambda l}{2}\right)}$$ (6)

where $\lambda =\sqrt{\frac{2k}{Et}}=\sqrt{\frac{2G}{hEt}}$. It can be seen from Eq 6 that the shear stress distribution in the shear layer is related to not only the mechanical parameters of rock matrix and the imposed external shear load *P*, but also the thickness *h* and the length *l* of the shear layer.

When $x=\pm \frac{l}{2}\tau$, *i*.*e*., at the left & right ends of the sample, the maximum shear stress can be calculated by Eq (7):

$$\tau_{max}=\frac{\lambda P}{2}coth\frac{\lambda l}{2}$$ (7)

The average shear stress in the sample shear layer can be determined by Eq (8):

$$\tau_{m}=\frac{1}{l}\int_{-\frac{l}{2}}^{\frac{l}{2}}\tau dx=\frac{P}{l}$$ (8)

The shear stress concentration coefficient is defined as follows:

$$K_{t}=\frac{\tau_{max}}{\tau_{m}}=\frac{\lambda l}{2}coth\frac{\lambda l}{2}$$ (9)

It can be seen from Fig 5, the yield stage of the direct shear test curve of the layered rock specimens can be basically ignored. As the horizontal shear load increases, the shear stress at both ends of the sample reaches the shear strength of the sample first, leading to cracks. With the growth of load, cracks gradually expand and penetrate into the middle part of the sample. When the shear stress concentration coefficient increases, the shear failure is more likely to occur at the shear stress concentrated area. Hence, the shear stress concentration coefficient could reflect the shear strength of rock specimens to a certain extent, and can also be applied to estimate the anisotropic characteristics of shear strength of layered rock specimens.

**Fig 7:**
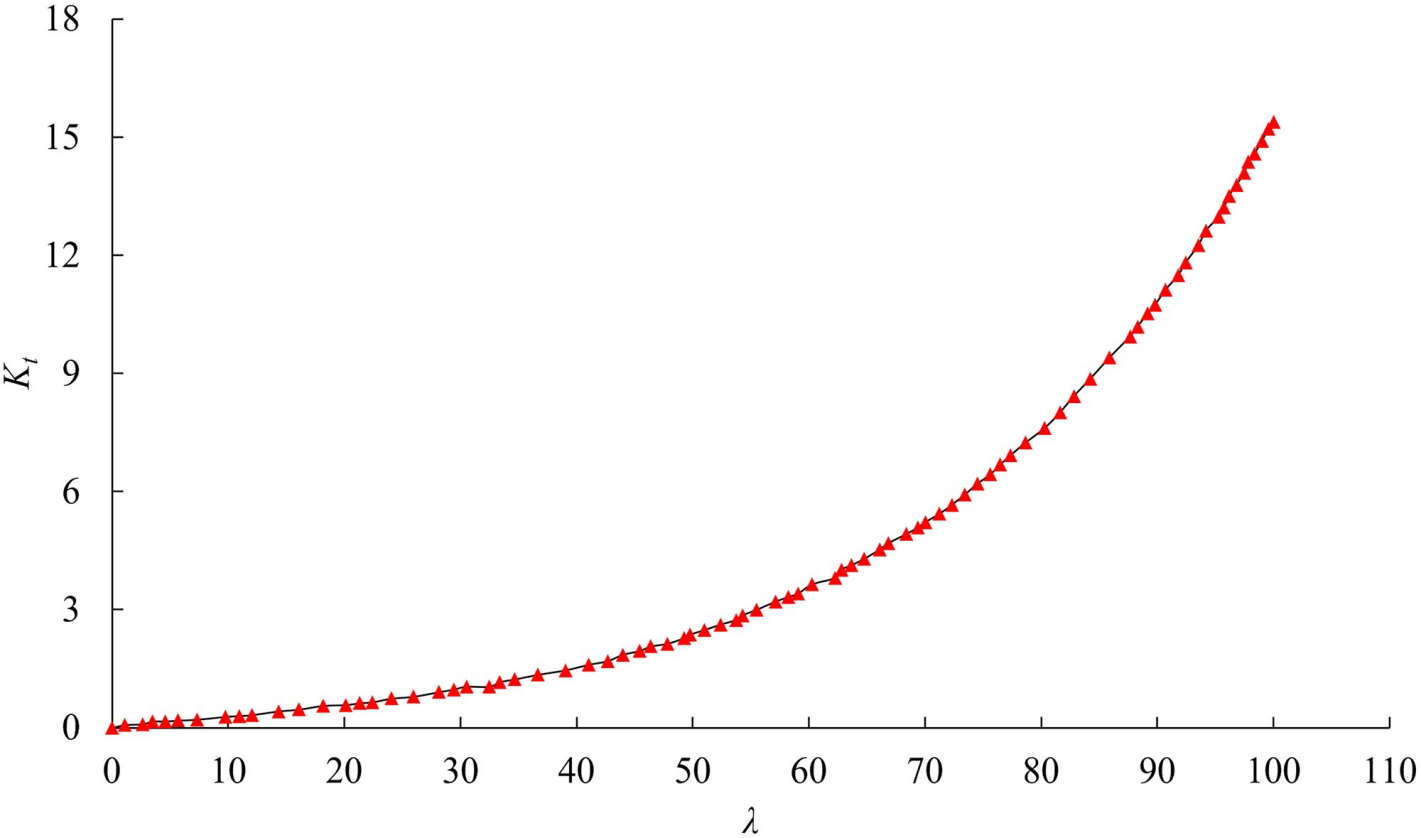
Curve of the shear stress concentration factor *K*_*t*_ changing with *λ*.

Because the shear stress concentration is easily generated at both ends of the sample shear layer, shear slip failure may occur at both ends of the shear layer under a certain axial stress. When the shear stress concentration coefficient becomes bigger, the shear stress at both ends of the shear layer will also increase under the same shear load. Therefore, the higher the shear stress concentration coefficient along the shear direction, the smaller the macroscopic shear strength of the specimen. According to Heng *et al*. (2014) [27], when the unit length *l* is considered, the change curve of shear stress concentration coefficient *K*_*t*_ with the growth of *λ* is displayed in Fig 7. We can see that the shear stress concentration coefficient rises gradually with the growth of *λ*, and the two are positively correlated.

Considering that $\lambda =\sqrt{\frac{2G}{hEt}}$, and *G*, *h*, *t* are assumed to remain constant for layered rock specimens. Namely, *λ* decreases with the growth of the elastic modulus of layered rock specimens, and the two indices show a negative correlation. Simultaneously, it is clear that the shear stress concentration coefficient *K*_*t*_ is negatively correlated with the elastic modulus of layered rock mass *E*. For the layered shale used in this study, the elastic modulus of shale specimen gradually increases as the bedding plane dip angle rises. The theoretical formula [38] for calculating the elastic modulus of shale samples with various bedding plane dip angles is shown as follows:

$$\frac{1}{E_{\theta }}=\frac{cos^{4}\theta }{E}+\frac{sin^{4}\theta }{E^{\prime }}+\frac{sin^{2}2\theta }{4}\left(\frac{1}{E}+\frac{1}{E^{\prime }}\right)$$ (10)

where *E* is the elastic modulus parallel to the bedding planes, and *E*′ is the elastic modulus perpendicular to the bedding planes.

By substituting Eq 10 into the expression about *λ*, it can be rewritten as follows:

$$\lambda =\sqrt{\frac{2G}{hE_{\theta }t}}=\sqrt{\frac{2G}{ht}\left[\frac{cos^{4}\theta }{E}+\frac{sin^{4}\theta }{E^{\prime }}+\frac{sin^{2}2\theta }{4}\left(\frac{1}{E}+\frac{1}{E^{\prime }}\right)\right]}$$ (11)

Besides, it is worth noting that the direct shear strength of layered rock specimen gradually increases as the bedding orientation rises. According to Eq 7, the maximum shear stress in the shear layer at both ends also exhibits the anisotropic characteristics influenced by the bedding orientation. Additionally, for the layered rock specimens subjected to normal stress in this study, the maximum shear stress progressively increases with the growth of the bedding plane angle.

### 4.2 Anisotropic shear strength

Table 2 shows the direct shear strengths of layered rock mass samples under various normal stresses. Because of the existence of weak bedding planes, the direct shear strength of the specimens with different bedding dip angles exhibits apparent anisotropy when they are subjected to various normal stresses. When the normal stress remains the same, the sample with the minimum layer inclination of 0° has a relatively low bond strength because of the shear failure mode of the specimen and the weak plane of the bedding plane. We can see from Table 2 that when suffering the same normal stress, the maximum shear strength sample has a bedding dip angle of 75° rather than 90°. The main reason is that the specimen with the bedding dip angle of 75° is not subject to the pure shear failure, but the shear slip along the weak bedding planes as well as the shear composite failure mode of rock material cross the weak bedding planes. However, when the inclination angle of the beddings rises up to 90°, the pure shear failure of rock material occurs. For the sample with the bedding dip angle of 0°~45°, the shear strength of the specimen increases gradually under the same normal stress. Under the condition of direct shear, the failure mode of transversely isotropic rock material is more complex than the isotropic rock material because the later mainly presents the composite failure mode of the shear slip of weak bedding planes & the shear of rock material. Actually, this can be recognized as the main reason why the direct shear strength of transversely isotropic rock material exhibits the anisotropic characteristics.

**Table 2:** Direct shear strength of the layered rock samples with various bedding orientations under varying normal stresses.

Normal stress (MPa)	Shear strength of the samples with various bedding orientations (MPa)						
	0°	15°	30°	45°	60°	75°	90°
2.5	7.10	10.22	10.94	11.86	11.68	15.52	15.26
5.0	7.83	12.07	11.61	13.49	12.78	16.99	16.56
7.5	10.00	13.32	12.04	15.59	13.34	18.45	17.07
10.0	10.87	13.64	12.33	16.35	14.41	18.70	17.62

### 4.3 Anisotropy of cohesion and internal friction angle

**Fig 8:**
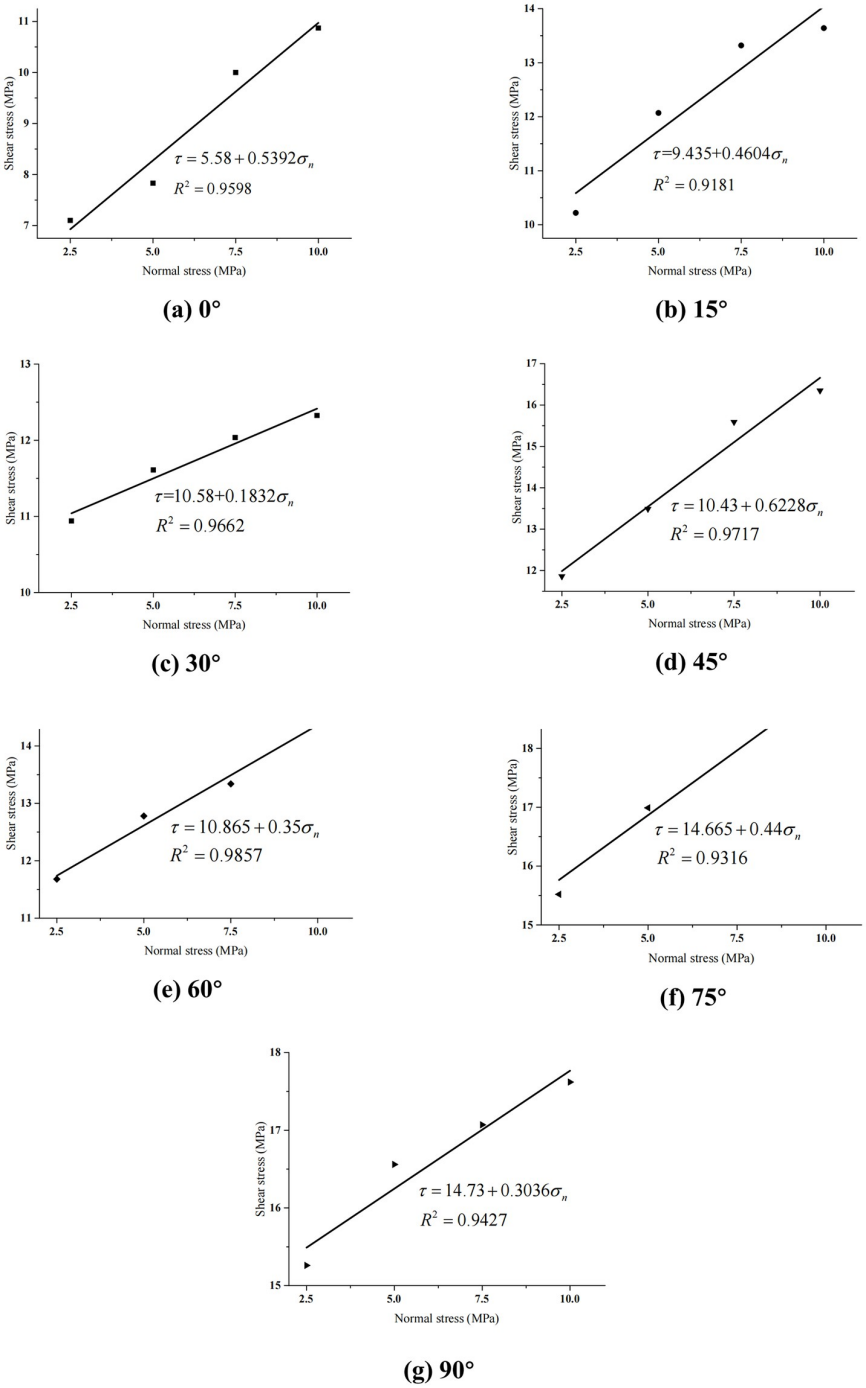
Correlation between shear strength and normal stress of the layered rocks.

According to the direct shear strength of the rock specimens with different bedding plane inclinations under varying normal stresses, the correlation between shear strength and normal stress is established as shown in Fig 8. Based on the theoretical basis of the Mohr-Coulomb criterion as described by Eq 1, the shear strength indices, *i*.*e*., cohesion and internal friction angle, of the rock specimens with various bedding orientations can be gained through the linear fitting. For the specimen with a bedding plane dip angle of 0°, the pure shear fracture occurs at the weak bedding surfaces. Therefore, the macroscopic shear strength of the sample is governed by the shear strength indices (*i*.*e*., cohesion *c*_*w*_ and internal friction angle *φ*_*w*_) of the weak bedding surfaces. Besides, the cohesion and internal friction angle of weak bedding planes are obviously smaller than the specimens with the other bedding plane dip angles. For the layered shale, the bedding plane is generally a weak structural plane, and its bond strength is greatly lower than the rock material. Therefore, the weak bedding plane is often destroyed before the rock material.

**Fig 9:**
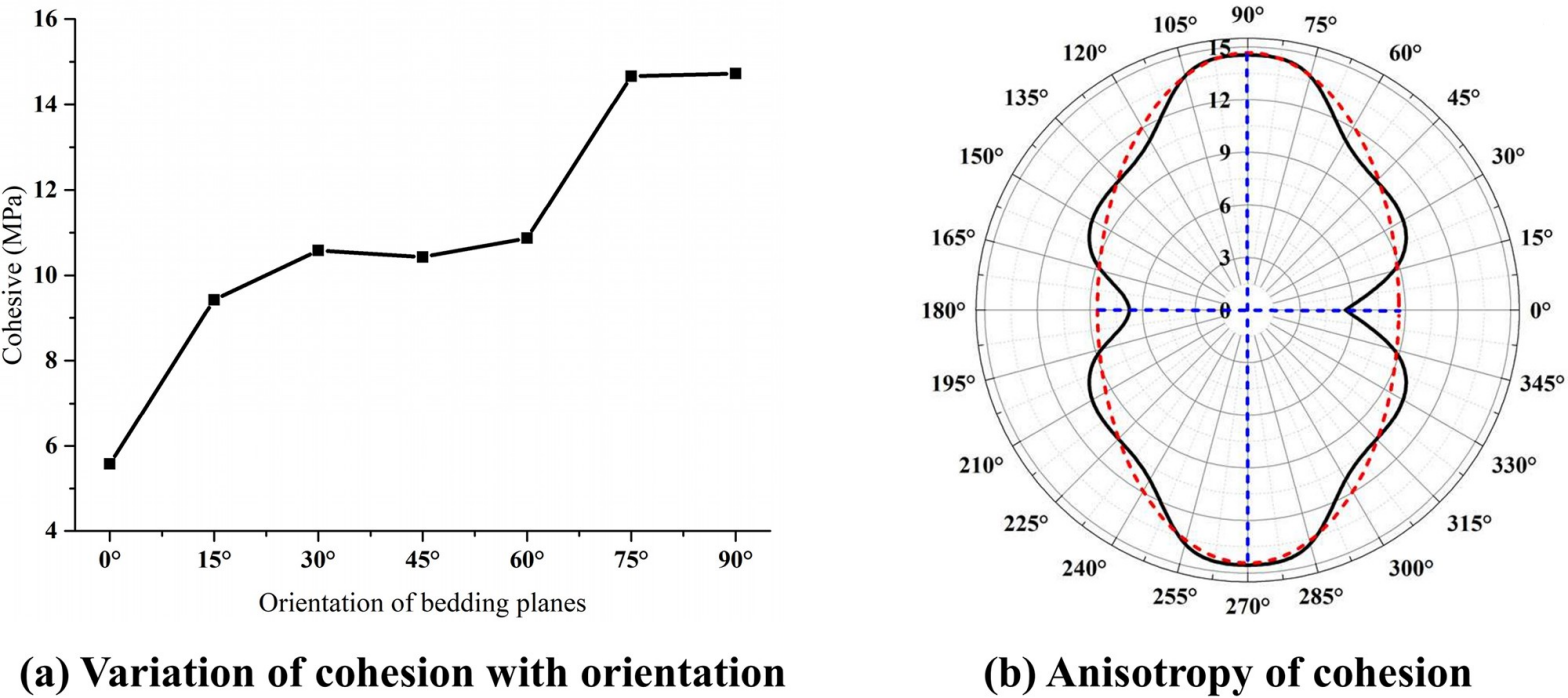
Cohesion of the layered rock samples with various inclination angles. (a) Variation of cohesion with orientation. (b) Anisotropy of cohesion.

Fig 9 shows that the cohesive force curves of the layered rock mass specimens changes as the bedding dip angle increases. It can be seen that with the growth of bedding plane angle, the cohesive force of the samples will gradually increase. When the surface inclination rises from 0° to 15°, the sample adhesion rate increases rapidly. This phenomenon is mainly because the failure mode of the sample has changed. If the bedding plane dip angle rises from 15° to 60°, the cohesion of the specimens increases gradually. However, the increasing rate is slow. This is mainly because the fracture patterns of the samples are basically the same, *i*.*e*., the composite failure modes of the shear slip along the bedding and the tensile fracture of rock material. If the bedding plane dip angle rises from 60° to 75°, the growth rate of the specimen cohesion increases rapidly, and the fracture pattern of samples varies gradually to the shear failure of rock materials. If the dip angle of the bedding plane rises from 75° to 90°, the sample cohesion increases slightly, which is because the fracture pattern of the specimen basically does not change, and the shear failure of the rock matrix is the dominant. We can see from Fig 9(b) that under direct shear, the cohesive force of the layered rock specimens shows the typical anisotropy characteristics, which can be fitted by the red elliptic dotted lines and simplified to the transverse isotropy. If the dip angle of bedding plane equals 0°, the cohesion sample reaches minimum, while it reaches maximum when the dip angle of bedding plane reaches 90°.

**Fig 10:**
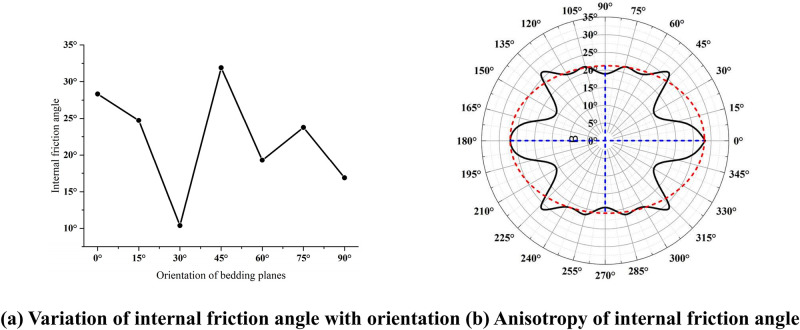
Internal friction angle of the layered rock samples with various inclination angles. (a) Variation of internal friction angle with orientation. (b) Anisotropy of internal friction angle.

Fig 10 shows the variation and anisotropy of the internal friction angles of the layered rock specimens with various bedding dip angles. It is clear that with the change of the dip angle of the bedding planes, the internal friction angle of the specimen decreases first, then increases and later decreases. Although the fluctuation of the internal friction angle changing with the bedding dip angle is not significant, it has the obvious anisotropy characteristics. Simultaneously, we can see from Fig 10(b) that the internal friction angle of the layered rocks can be also fitted by the red elliptic dotted line and simplified to the transverse isotropy.

## 5 Discussions

Nova and Sacchi (1979) [39] obtained the failure conditions of layered rocks suffering triaxial compression through analytical solutions, which can be expressed by Eq (12):

$$\frac{\sigma_{1}-\sigma_{3}}{2}={\left(f_{1\theta }f_{2\theta }\right)}^{1/2}-sin2\theta_{m}\left[\frac{2\mu_{t}\sigma_{r}\left(\beta -1\right)}{2}+\frac{c_{t}\left(\alpha -1\right)}{2}\right]$$ (12)

where *σ*_1_ and *σ*_3_ are the maximum and minimum principle stresses, respectively; *θ*_*m*_ is the angle between bedding plane and maximum principle stress; *f*_1*θ*_, *f*_2*θ*_ and *σ*_*r*_ are the functions of the principle stresses, respectively; *μ*_*t*_, *c*_*t*_, *α* and *β* are the strength parameters of layered rocks, respectively.

**Fig 11:**
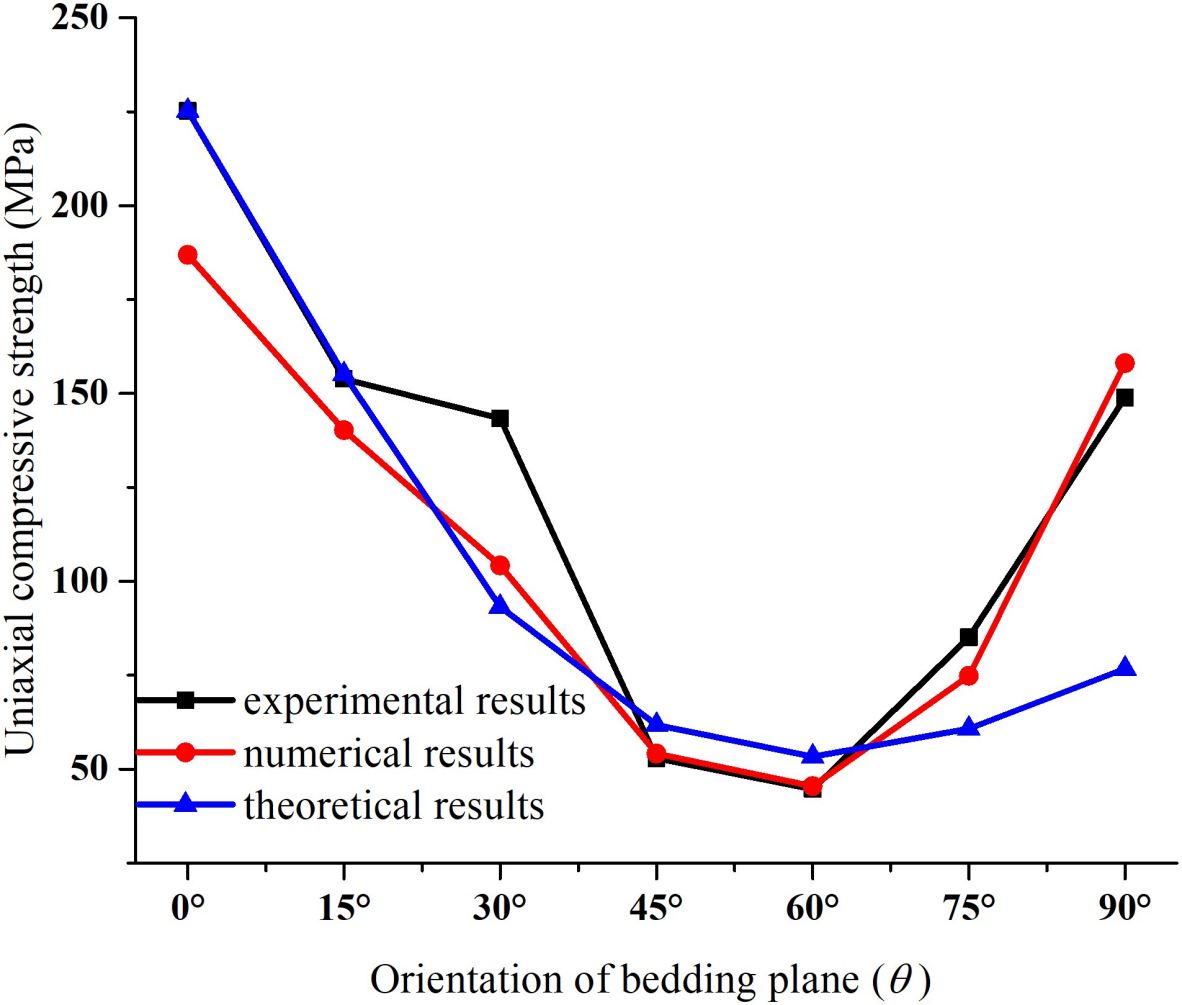
The uniaxial compressive strengths of layered rocks obtained by test, simulation and theoretical analysis.

On the basis of the uniaxial compression experiment and triaxial compression numerical modelling of the layered rocks [40], the parameters *c*_*t*_ = 20.03, 2*μ*_*t*_ = 0.685463, *α* = 3.6 and *β* = 0.015 can be determined. According to Eq 12, the variation rule of the compression strength affected by various confining pressures and bedding dip angles can be obtained. The comparison of the uniaxial compression experiment, theoretical analysis and numerical simulation is shown in Fig 11. It is clear from Fig 11 that the uniaxial compressive strength of layered rock mass predicted by theoretical analysis is similar to the experimental and simulated data. Meanwhile, the uniaxial compressive strength decreases first and then grows up as the dip angle of the bedding plane *θ* increases, showing a U-shaped variation. When the bedding plane dip angle equals 30° & 90°, the uniaxial compression strength predicted by theory is smaller than the experimental and simulated values. On the whole, the theoretically predicted values agree with the experimental and numerical data, and their variation rules are basically consistent.

Figs 9(a) and 10(a) show the changing curves of the cohesion and internal friction angle of the layered rock masses with different layer surface inclinations under the direct shear condition, respectively. Gao *et al*. (2023) [41] analyzed the corresponding changing curves under the triaxial compression condition. Their comparison implies that although the variations of the cohesive force and internal friction angle are different, they all show the obvious anisotropic characteristics. By analyzing the failure patterns of the samples, it can be found that the failure modes under direct shear are obviously different from those under triaxial compression [41]. This phenomenon is caused by their different failure patterns. Considering that rock mass is generally suffering the complex three-dimensional stress state in rock engineering, it is more suitable to adopt the changing laws of cohesion and internal friction angle and the anisotropy characteristics of substratum rock mass under triaxial compression condition for actual engineering projects.

According to the results of theoretical analysis and numerical simulation, the cohesive force of layered rock mass can be fitted when the internal friction angle is assumed to remain unchanged. For the U-shaped curve of cohesion changing with the inclination of bedding plane, it can be fitted in the form of trigonometric function and expressed as follows:

$$\frac{1}{c_{\theta }}=\frac{sin^{4}\theta }{c_{90}}+\frac{cos^{4}\theta }{c_{0}}+\left(\frac{c_{0}}{c_{90}}-\frac{1}{2}\right)sin^{2}\theta cos^{2}\theta$$ (13)

where *c*_*θ*_ is the cohesion of the sample with bedding orientation of *θ*; *c*_0_ and *c*_90_ are the cohesion of the sample with bedding orientation of 0° and 90°, respectively. If the cohesion in the Mohr-Coulomb criterion is replaced by Eq 13, the modified Mohr-Coulomb criterion can be obtained.

## 6 Conclusions

In this study, the numerical layered rock specimens are established. Then, the shear failure modes, shear stress vs. shear displacement curves and shear strengths are modelled under different shear conditions. Meanwhile, the anisotropic features of layered rocks are analyzed theoretically, and the shear mechanical parameters under triaxial conditions are investigated and compared with the simulated data. Furthermore, the empirical fitting formula of shear strength indexes and the modified Mohr-Coulomb failure criterion are discussed. The main conclusions are as follows:

1. Under the condition of direct shear, the failure patterns of layered rock samples can be classified into three categories, *i*.*e*., the shear failure along bedding planes, the shear failure across bedding planes, and the combination of shear failure across bedding planes and tensile failure along bedding planes.
2. Under the condition of direct shear, the cohesion of layered rock specimens increases gradually with the growth of the dip angle of bedding planes, and the internal friction angle of the samples drops first, then increases and later decreases with the growth of the dip angle of bedding planes, indicating the typical anisotropy characteristics. Simultaneously, when subjected to triaxial compression, the cohesion of layered rock specimens decreases first, but then increases with the growth of the dip Angle of bedding planes, showing a typical ‘U’ shaped curve. However, the internal friction angle of the specimens increases gradually with the growth of the dip angle of bedding planes.
3. According to the theoretical and numerical results, the empirical fitting formula characterizing the shear strength of layered rocks suffering triaxial compression is provided, and the modified Mohr-Coulomb criterion reflecting the rock anisotropy is proposed.
